# SfgA Renders *Aspergillus flavus* More Stable to the External Environment

**DOI:** 10.3390/jof8060638

**Published:** 2022-06-16

**Authors:** Xiao-Yu Yuan, Jie-Ying Li, Qing-Qing Zhi, Sheng-Da Chi, Su Qu, Yan-Feng Luo, Zhu-Mei He

**Affiliations:** 1 The Guangdong Province Key Laboratory for Aquatic Economic Animals, School of Life Science, Sun Yat-sen University, Guangzhou 510275, China; yuanxy8@mail2.sysu.edu.cn (X.-Y.Y.); lijieyingjy@126.com (J.-Y.L.); zhiqq3@mail.sysu.edu.cn (Q.-Q.Z.); sd_chi@163.com (S.-D.C.); qusu@mail2.sysu.edu.cn (S.Q.); 2 College of Agriculture and Biology, Zhongkai University of Agriculture and Engineering, Guangzhou 510225, China; 3 Guangdong Jinyinshan Environmental Protection Technology Co., Ltd., Guangzhou 510705, China; kelyna@foxmail.com

**Keywords:** *Aspergillus flavus*, *sfgA*, sclerotia, aflatoxin, conidiation, secondary metabolism, stress response, RNA-seq

## Abstract

*sfgA* is known as a key negative transcriptional regulator gene of asexual sporulation and sterigmatocystin production in *Aspergillus nidulans*. However, here, we found that the homolog *sfgA* gene shows a broad and complex regulatory role in governing growth, conidiation, sclerotia formation, secondary metabolism, and environmental stress responses in *Aspergillus flavus*. When *sfgA* was deleted in *A. flavus,* the fungal growth was slowed, but the conidiation was significantly increased, and the sclerotia formation displayed different behavior at different temperatures, which increased at 30 °C but decreased at 36 °C. In addition, *sfgA* regulated aflatoxin biosynthesis in a complex way that was associated with the changes in cultured conditions, and the increased production of aflatoxin in the ∆*sfgA* mutant was associated with a decrease in sclerotia size. Furthermore, the ∆*sfgA* mutant exhibited sensitivity to osmotic, oxidative, and cell wall stresses but still produced dense conidia. Transcriptome data indicated that numerous development- and secondary-metabolism-related genes were expressed differently when *sfgA* was deleted. Additionally, we also found that *sfgA* functions downstream of *fluG* in *A. flavus*, which is consistent with the genetic position in FluG-mediated conidiation in *A. nidulans*. Collectively, *sfgA* plays a critical role in the development, secondary metabolism, and stress responses of *A. flavus,* and *sfgA* renders *A. flavus* more stable to the external environment.

## 1. Introduction

*Aspergillus flavus* is an opportunistic filamentous fungus which infects agricultural crops such as maize, peanuts, and cotton [1]. *A. flavus* spores germinate on crops and foods and produce detrimental secondary metabolite mycotoxins, including aflatoxins, which are harmful fungal mycotoxins that cause carcinogenesis in animals and humans, and thus, enormous economic losses [2]. Therefore, exploration of the regulatory mechanism of the development and secondary metabolism of *A. flavus* is vital to control aflatoxin pollution.

The *A. flavus* reproductive cycle involves an asexual growth phase and sexual developmental phase [3,4]. During asexual growth, *A. flavus* differentiates into a variety of structures including spores, which are crucial for genome protection, survival, and proliferation. Additionally, asexual sporulation causes the production of mycotoxin sterigmatocystin or other secondary metabolites [5,6]. In *A. flavus*, the formation of asexual spores is closely related to the production of aflatoxin and the formation of sclerotia [7,8]. Sclerotium, a structure formed in a critical developmental stage, mainly infects crops and responds to harsh environmental conditions. Extensive studies have reported that the development of sclerotia is closely related to the synthesis of secondary metabolites, and many of them, such as aflatoxins, have been found in sclerotia [9,10]. Hence, morphological development and the secondary metabolism are generally considered to be linked with each other or co-regulated in *A. flavus* and some other fungal species [11,12,13].

Studies focusing on the conidiation regulatory mechanism in model fungal *Aspergillus nidulans* have been conducted and have provided insight into asexual development and the secondary metabolism [14,15]. According to a report by Park et al., there are three genes, *brlA*, *abaA,* and *wetA,* constructing the central developmental pathway of conidiation in filamentous fungi [16]. Later studies have identified various upstream developmental activators, FluG and Flbs (FlbA, B, C, D, and E), which can activate the essential conidiophore developmental regulator BrlA [17,18]. In addition, FluG and FlbA are interdependent, thereby inhibiting proliferation mediated by the heterotrimeric G protein composed of FadA and SfaD::GpgA [19,20,21]. FluG has been considered as the most upstream regulator that regulates the growth and development in *Aspergillus*. However, the function of *fluG* and the mechanism of conidiation vary between in *A. nidulans* and *A. flavus*. The *fluG* gene is necessary for the production of conidia and the synthesis of the carcinogenic mycotoxin sterigmatocystin in *A. nidulans*, while the absence of *fluG* in *A. flavus* does not affect the formation of aflatoxin [7,22]. These observations suggest that these two species of *Aspergilli* possess both conserved and divergent signaling pathways associated with the regulation of asexual sporulation and secondary metabolism [23].

According to previous studies, *sfgA* functions downstream of *fluG* but upstream of transcriptional activator genes (*flbA*, *flbD*, *flbC*, *flbB*, and *brlA*) necessary for normal conidiation and sterigmatocystin biosynthesis [24]. In *A. nidulans*, *sfgA* was reported to be the key suppressor of *fluG,* because there was conidia formation and sterigmatocystin production in the *fluG*::*sfgA* double-deletion strains compared with no conidiation in the *fluG* deletion mutant [25]. Although SfgA is conserved among most *Aspergillus* species, which was predicted to be a transcription factor containing the Gal4-type Zn(II)_2_Cys_6_ domain [25], sequence conservation does not guarantee the conservation of the functions in other *Aspergillus* spp.

The aim of the present work was to evaluate the functions of the homolog gene *sfgA* in regulating the development and secondary metabolism of *A. flavus.* To examine the role of SfgA, an *sfgA* deletion mutant (∆*sfgA*) strain was generated, and its phenotypes and transcriptome were analyzed. Our results demonstrate that *sfgA* appeared to be functioning as a global regulator in the development and secondary metabolism of *A. flavus*. This study should contribute to the understanding of the regulatory networks that control fungal development and the production of secondary metabolites.

## 2. Materials and Methods

### 2.1. Fungal Strains and Media

*Aspergillus flavus* TXZ21.3 (Δ*ku70*, Δ*argB*, and *pyrG-*) was used as the parental strain to construct ∆*sfgA* and OE*sfgA* mutant strains, and TJES19.1 (Δ*ku70* and *pyrG-*) was adopted as a control to exclude the interfering factors of supplemental uracil and uridine in media [10]. The glucose minimum medium (GMM, 10 g/L glucose, 6 g/L NaNO_3_, 1.52 g/L KH_2_PO_4_, 0.52 g/L KCl, 0.52 g/L MgSO_4_·7H_2_O, and 1 mL of trace elements, pH 6.5), yeast extract–sucrose (YES, 20 g/L yeast extract and 60 g/L sucrose, pH 5.8), yeast extract–glucose (YGT, 5 g/L yeast extract, 20 g/L glucose, and 1 mL of trace elements), potato dextrose agar (PDA, Difco), and potato dextrose broth (PDB, Difco) were used for morphological observations. For transformation, YGT and sorbitol minimal medium (SMM, 10 g/L glucose, 6 g/L NaNO_3_, 1.52 g/L KH_2_PO_4_, 0.52 g/L KCl, 0.52 g/L MgSO_4_·7H_2_O, 1 mL of trace elements, and 1.2 M sorbitol, pH 6.5) were used. In addition, 1 g/L uracil and 1 g/L uridine (denoted as “UU” when necessary) or 1 g/L arginine (denoted as “A” when necessary) were adopted to grow auxotroph.

### 2.2. Fungal Transformation

*A. flavus* protoplast preparation and transformation were carried out according to the protocol of He et al. [26], with some modifications that are described as follows. Briefly, 10^8^ spores were inoculated into 100 mL of YGTAUU liquid medium and incubated at 30 °C, 150 rpm for 11 h. Then, the mycelia were harvested and washed with sterile water through centrifugation at 11,000 rpm for 5 min. Protoplasts were prepared with a protoplast solution composed of 20 mM NaH_2_PO_4_, 20 mM CaCl_2_, 200 µL of *β*-glucuronidase (85,000 U/mL, Sigma, MO, USA), 200 mg of lysing enzymes from *Trichoderma harzianum* (Sigma), and 50 mg of Driselase from *Basidiomycetes* sp. (Sigma) in 1.2 M NaCl. Protoplasting was performed at 80 rpm and 30 °C for 4–6 h. After transformation, the protoplasts were plated on SMM medium plus appropriate supplements.

### 2.3. Fungal Physiology Experiments

For the morphological observation of colonies, 1 µL of conidia suspension containing approximately 10^3^ conidia was point-inoculated on GMMUU and YGTUU solid plates and cultured under light for 5 d at 30 °C. For the spore germination assay, *A. flavus* conidia (10^6^ spores) were inoculated in 10 mL of PDBUU media with coverslips at 30 °C. The morphology of germinated conidia and hyphae was visualized under a light microscope (Magnification, 200×) at different time intervals. For the analysis of conidial production, 5 mL of conidia suspension (10^6^ spores/mL) dispersed in molten PDBUU medium supplemented with 0.7% agar was overlaid on the PDAUU plates (1.5% agar). Sclerotia production was measured as previously described [27] by counting sclerotia from GMMUU culture plates after incubation for 14 d at 30 °C and 36 °C under darkness. Sclerotia size was photographed using a stereo microscope (SteREO Lumar.V12, ZEISS; magnification: 50×). For the stress test, PDAUU solid plates were supplemented with the following agents: 1.2 M NaCl, 1.2 M KCl and 1.5 M sorbitol for hyperosmotic stress, 6 mM H_2_O_2_, 1.8 mM t-BOOH for oxidative stress, and 0.2 mg/mL congo red for cell wall stress.

### 2.4. Examination of Aflatoxin and Kojic Acid

Aflatoxin B1 (AFB1) production was measured via modified thin-layer chromatography (TLC), as previously described [28]. Each *A. flavus* strain was inoculated on GMMUU, YESUU, YGTUU, and PDBUU at 30 °C and 36 °C, and the same weight of mycelia or the same number of sclerotia was collected for AFB1 extraction. AFB1 on the TLC plates could be visualized using a fluorescent detector with a UV wavelength of 254 nm, and then, the aflatoxin production was quantified using Image J software. Standard AFB1 was purchased from Sigma. Kojic acid production was determined using the colorimetric method, as previously reported [13]. Briefly, *A. flavus* strains were cultured on PDAUU, YGTUU, and YESUU supplemented with 1 mM FeCl_3_ for 36 h at 30 °C and 36 °C. Kojic acid forms a chelated compound with ferric ions and subsequently generates a red color, allowing for a qualitative comparison between different strains.

### 2.5. Catalase Activities Measurement

Around 50 mg of mycelia cultured in PDBUU medium for 24 h was suspended in 500 µL of extracting solution, and then, samples were centrifuged at 8000× *g* for 10 min at 4 °C, and the supernatant was used to measure the catalase activity according to the manufacturer’s instructions. The catalase assay kit (BC0205) was purchased from Solarbio (Beijing, China).

### 2.6. qRT-PCR Analysis

Spores were inoculated in 30 mL of PDBUU to a final concentration of 3 × 10^5^/mL and incubated at 30 °C with shaking (200 rpm) for 48 h. Total RNA was extracted from the harvested mycelia using Trizol Reagent (Invitrogen, Carlsbad, CA, USA), and cDNA was synthesized from 1 µg of RNA using the HiScript α Q RT SuperMix cDNA Synthesis kit (Vazyme, Nanjing, China). The qRT-PCR assay was performed using the LightCycler^®^ 480 (Roche, Basel, Switzerland) with SYBR Green (Vazyme, Nanjing, China) detection, as described previously [27]. Gene expression levels were normalized (2^−∆∆Ct^ analysis) to *A. flavus β*-actin gene expression levels. All analyses were performed in triplicate. The primers used for qPCR are listed in Supplementary Table S2.

### 2.7. RNA Sequencing and Data Analysis

RNA samples from three independent biological repeats from *A. flavus* of the TJES19.1 control and ∆*sfgA* mutant were prepared. Strains were propagated on GMMUU at 30 °C and 36 °C for 48 h, and mycelia were harvested immediately for RNA extraction using Trizol Reagent (Invitrogen). The quality and quantity of isolated RNA were determined using an Agilent 2100 bioanalyzer system, and RNA integrity numbers (RINs) were calculated. RNA samples with a RIN ≥ 8 were used for sequencing library preparation with an Illumina TruSeq RNA Sequencing Kit. The libraries were sequenced on an Illumina Hiseq2500 system (Oebiotech, Shanghai, China).

### 2.8. Statistical Analysis

All statistical analyses were performed using GraphPad Prism (version 8.0; GraphPad Software), and *p* < 0.05 was considered a significant difference.

## 3. Results

### 3.1. Identification of SfgA in A. flavus

**Figure 1:**
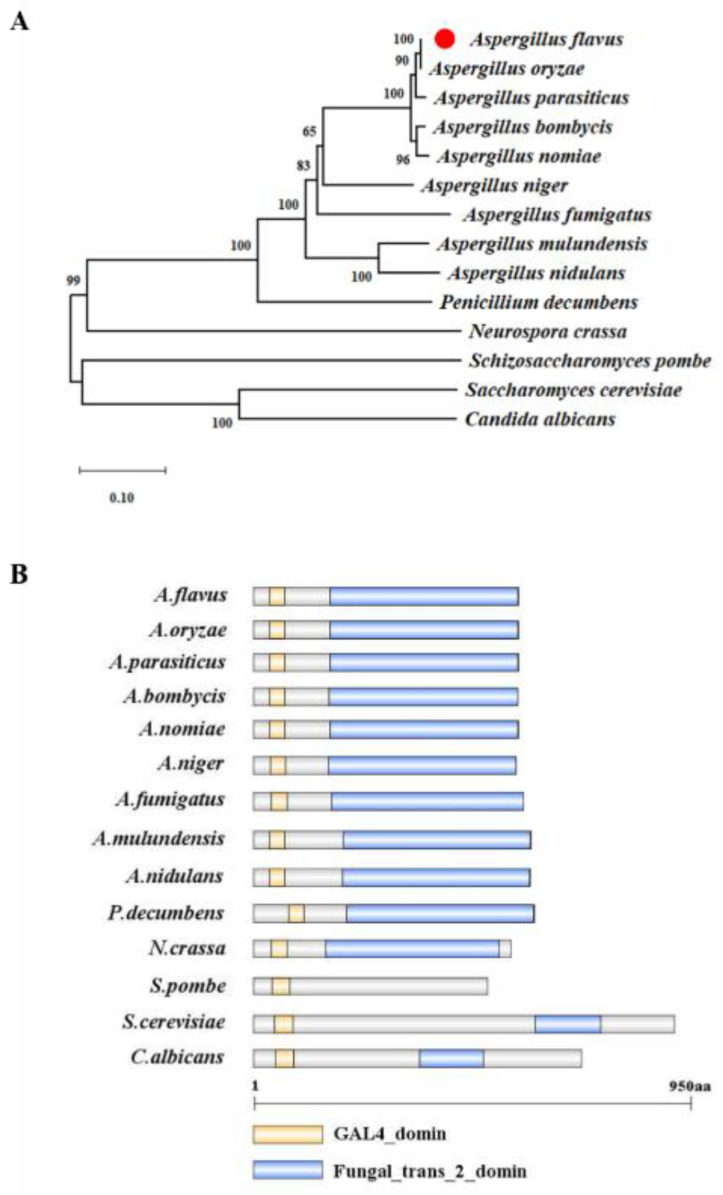
Summary of SfgA from different fungi. (**A**) A phylogenetic tree of the SfgA homologs identified in different species including *A. flavus* NRRL3357 (AFLA_005520). The tree was generated using MEGA 7 software with neighbor-joining and bootstrap method. (**B**) Domain analysis of the SfgA homologs in species. Protein structure was characterized using SMART and drawn using DOG 2.0 software.

To identify the ortholog of SfgA, the *A. flavus* NRRL3357 genome was screened by using the protein sequence of the model organism *A. nidulans* SfgA (XP_681398.1). XP_041146550.1 in *A. flavus* was 64% identical to *A. nidulans* SfgA by protein homology. Sequence alignment results showed that the *A. flavus* SfgA protein displayed 100% similarity to *Aspergillus oryzae* and 98% similarity to *Aspergillus parasiticus*. The phylogenetic analysis indicated that the SfgA protein is evolutionarily conserved in *Aspergillus* species (Figure 1A). The *A. flavus sfgA* (AFLA_005520) open reading frame (ORF) was predicted to consist of 1882 nucleotides, with two introns, and encodes a putative C6 transcription factor (SfgA) containing 575 amino acids (aa). Additionally, the predicted *A. flavus* SfgA harbors a GAL4 domain (residues 35–68 aa) and a fungal specific transcription factor domain (residues 166–574 aa). The structural analysis of SfgA proteins from several species showed that all analyzed fungi share a highly conserved GAL4 domain (Figure 1B).

**Figure 2:**
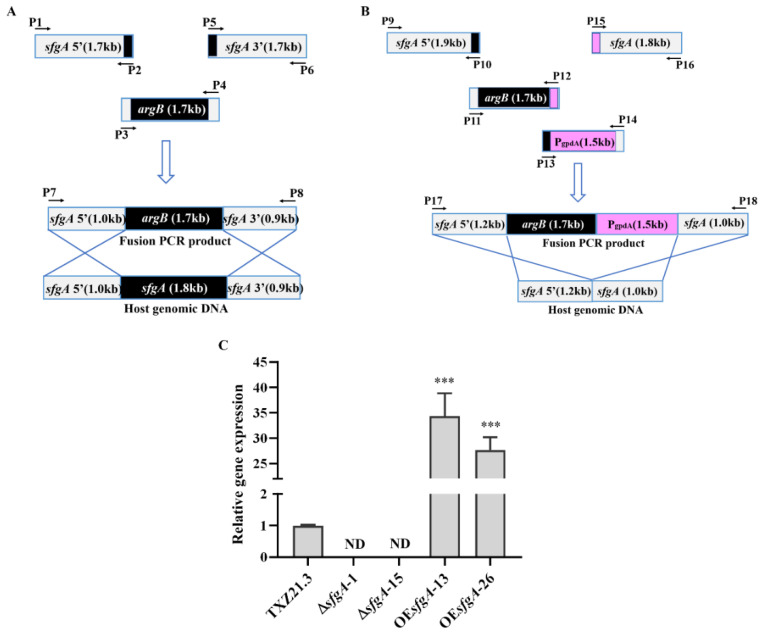
Generation of the ∆*sfgA* and OE*sfgA* mutants in *A. flavus*. (**A**,**B**) The scheme of *sfgA* deletion and over-expression strategy, respectively. (**C**) qPCR analysis for the *sfgA* gene expression in Δ*sfgA* and OE*sfgA* strains. TXZ21.3 (Δ*ku70*, Δ*argB*, *pyrG^−^*) is the transformation recipient strain. ND: not detected. *** *p* ≤ 0.001.

To test the potential biological function of *sfgA* in *A. flavus*, the *sfgA* deletion mutant (∆*sfgA*) and over-expression mutant (OE*sfgA*) were generated using the *argB* gene as a selection marker to complement the arginine auxotrophy of *Aspergillus flavus* TXZ21.3 (Δ*ku70*, Δ*argB*, and *pyrG-*). The schematic diagram of the homologous recombination strategy is shown in Figure 2A,B, and the primers used are shown in Supplementary Table S1. Afterward, the mutants were characterized by PCR (Supplementary Figure S1) and qPCR (Figure 2C) to confirm successful gene manipulation. Then, the loss-of-function strain Δ*sfgA*-1 and gain-of-function strain OE*sfgA*-13 were selected for further study.

### 3.2. sfgA Influences Growth and Conidiophore Development in A. flavus

**Figure 3:**
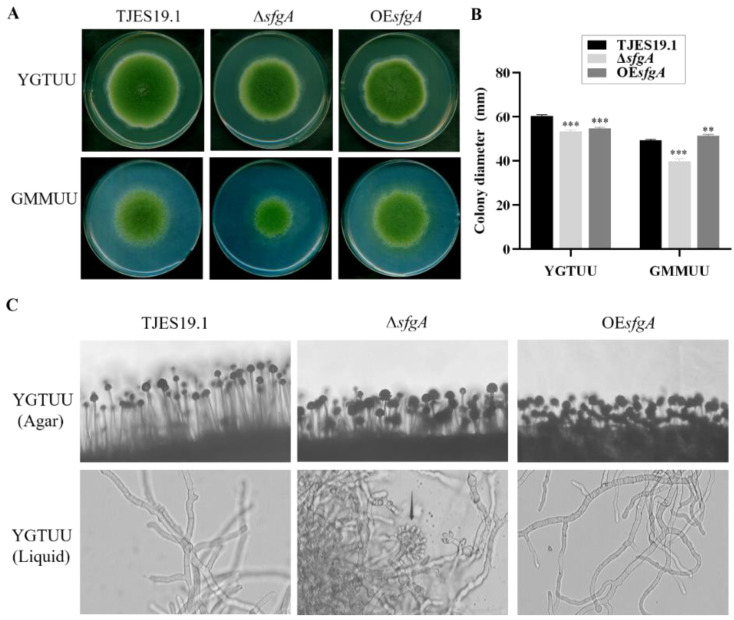
Fungal growth and conidiophore development of the ∆*sfgA* mutant. (**A**) The colony phenotype of TJES19.1, ∆*sfgA,* and OE*sfgA* strains point-inoculated on solid YGTUU and GMMUU media and propagated at 30 °C for 5 days. (**B**) Quantitative analysis of colony diameter shown in (**A**). ** *p* ≤ 0.01; *** *p* ≤ 0.001. (**C**) Conidia formation of all strains were observed under a light microscope (magnification, 200×) at 48 h post-inoculation onto solid YGTUU and 14 h after inoculation into liquid YGTUU, respectively.

To investigate the roles of *sfgA* in the fungal growth of *A. flavus*, the control (TJES19.1) and mutant strains were inoculated to YGTUU and GMMUU media and incubated for 5 d. As shown in Figure 3A,B, the results show that the colony growth of the ∆*sfgA* mutant was inhibited on both media when compared with TJES19.1 and OE*sfgA* strains. Moreover, microscopic observations revealed that conidiophore stipes were significantly shorter and denser in Δ*sfgA* and OE*sfgA* strains, resulting in a somewhat flat colony phenotype in contrast to the typical floccose appearance of the control strain (Figure 3C). Additionally, the absence of *sfgA* resulted in hyperactive conidiation, evidenced by the formation of conidiophores in liquid shake culture (Figure 3C). Additionally, 75% of Δ*sfgA* conidia germinated after 8 h of incubation, while only about 40% of control conidia were germinated (Supplementary Figure S2), indicating that *sfgA* may negatively regulate conidial germination.

*sfgA* was reported as the suppressor of *fluG* (SFGs) that bypasses the need of *fluG* in conidiation in *A. nidulans* [24]. To identify the relationship between FluG and SfgA in *A. flavus*, the Δ*sfgA*Δ*fluG* double mutant was generated. As shown in Supplementary Figure S3, both Δ*sfgA* and Δ*sfgA*Δ*fluG* mutations showed identical phenotypes in growth and conidiation, indicating that SfgA functions downstream of FluG in *A. flavus*, which is consistent with the genetic position of *sfgA* in the FluG-mediated conidiation in *A. nidulans* [25].

### 3.3. Roles of sfgA in Sclerotia Formation

**Figure 4:**
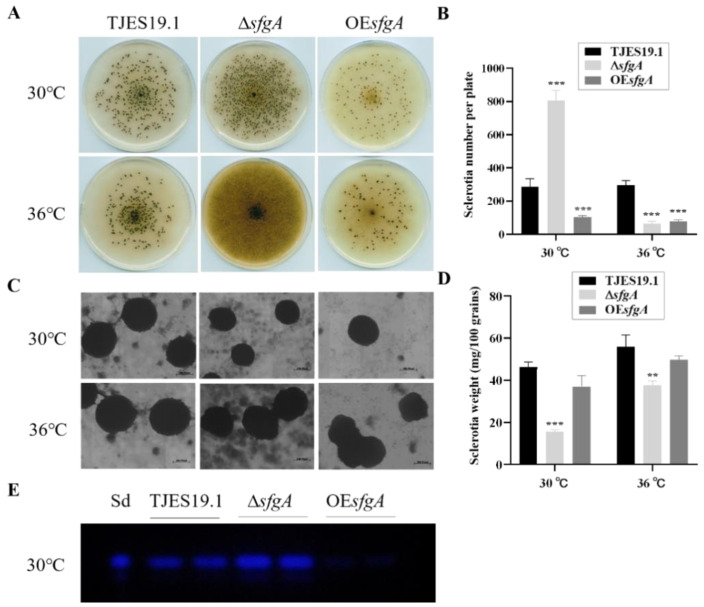
Sclerotia production of ∆*sfgA* in *A. flavus*. (**A**) Ethanol-washed colony photographs of TJES19.1, ∆*sfgA,* and OE*sfgA* strains propagated on solid GMMUU media under dark condition at 30 °C and 36 °C for 14 days. (**B**) Quantitative analysis of sclerotia of strains was conducted. (**C**) Stereo microscope photographs of sclerotia size of TJES19.1, ∆*sfgA* and OE*sfgA* strains; magnification: 50×. (**D**) Weight of 100 sclerotia of strains was measured. ** *p* ≤ 0.01; *** *p* ≤ 0.001. (**E**) Aflatoxin B1 produced in sclerotia cultured at 30 °C via thin-layer chromatography (TLC) analyses. Sd represents the AFB1 standard.

Sclerotia is commonly considered to be a survival structure of *A. flavus* against unfavorable conditions. To investigate the impact of the *sfgA* gene on sclerotia formation in *A. flavus*, the TJES19.1, Δ*sfgA*, and OE*sfgA* strains were point-inoculated on GMMUU medium and cultured at 30 °C and 36 °C for 14 d under dark conditions. After being sprayed with 75% ethanol, the number of sclerotia on each plate was counted. The result suggests that *sfgA* plays a complex role in sclerotia production in different conditions. A lack of *sfgA* significantly increased the production of sclerotia, and the over-expression of *sfgA* clearly decreased sclerotia production versus the control strain when cultured at 30 °C (Figure 4A,B). When cultured at 36 °C, to our astonishment, the sclerotial number of the Δ*sfgA* mutant declined sharply and was less than that of the control strain (Figure 4A,B). Furthermore, the sclerotia size produced in the Δ*sfgA* mutant at 30 °C was much smaller than that of the TJES19.1 and OE*sfgA* strains; however, this change was partly restored at 36 °C (Figure 4C). The weight of a single sclerotia in the Δ*sfgA* mutant was also lighter than that in the control strain at both temperatures (Figure 4D).

Interestingly, we found that the sclerotia formation, quantitated in size under different temperatures mediated by *sfgA*, was associated with aflatoxin accumulation. The accumulation of aflatoxin in the sclerotia of the Δ*sfgA* mutant was accompanied by a decrease in their sclerotia size (Figure 4E), which was previously reported in *A. parasiticus* by Chang et al. [29]. Aflatoxins were weakly produced in variant strains at 36 °C in GMMUU medium (data not shown), the variations of which were difficult to compare with.

### 3.4. sfgA Affects Secondary Metabolite Production of A. flavus

**Figure 5:**
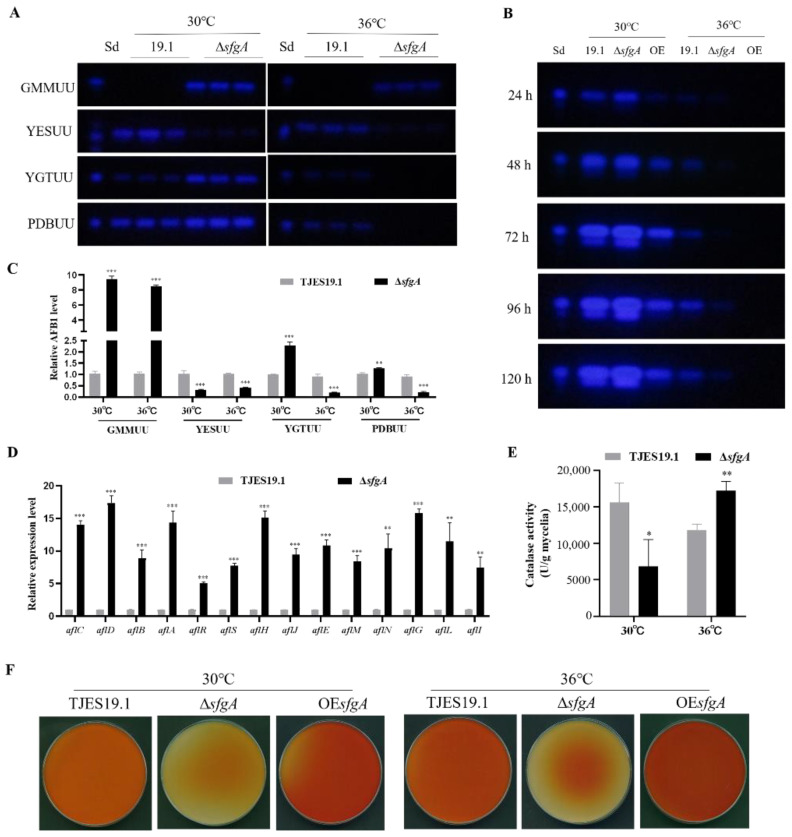
Aflatoxin and kojic acid production of ∆s*fgA* in *A. flavus*. (**A**) TLC analyses of AFB1 production of mycelia extracts cultured on GMMUU, YESUU, YGTUU, and PDBUU media, respectively. (**B**) TLC analyses of AFB1 production of culture extracts from PDBUU media. Sd represents the AFB1 standard. (**C**) Relative quantitative analyses of AFB1 from A by Image J software. (**D**) qPCR analysis of transcriptional levels of the aflatoxin cluster genes cultured in PDBUU at 30 °C for 48 h. Gene expression levels at each time point were normalized to *β*-actin by 2^−ΔΔ^CT analysis. * *p* ≤ 0.1; ** *p* ≤ 0.01; *** *p* ≤ 0.001. (**E**) Catalase activity of mycelia cultured in PDBUU at 30 °C and 36 °C for 24 h. (**F**) Determination of kojic acid production in solid PDAUU medium for 36 h via the colorimetric method.

Filamentous fungi can produce numbers of small bioactive molecules as part of their secondary metabolism, which is closely related with fungal developmental programs. The *sfgA* deletion strain showed different AFB1 levels, a crucial metabolite in *A. flavus*, in different culture conditions. When propagated on solid GMMUU media for 48 h, *sfgA* deletion resulted in elevated AFB1 levels at both 30 °C and 36 °C (Figure 5A,C). When cultured on solid YESUU media for 48 h, *sfgA* deletion resulted in reduced AFB1 levels at both 30 °C and 36 °C (Figure 5A,C). Inexplicably, when cultured in YGTUU media (Figure 5A,C) and PDBUU media (Figure 5A–C), the AFB1 level was increased in the ∆*sfgA* mutant at 30 °C, while the AFB1 level was decreased at 36 °C, from which it is evident that the mode of aflatoxin biosynthesis in Δ*sfgA* would vary in accordance with the external environmental factors. The results of qPCR (Figure 5D) showed that *sfgA* affected the aflatoxin production through regulating transcription in aflatoxin cluster genes. Furthermore, as the antioxidant enzyme catalase is linked with reactive oxygen species (ROS) stress response with aflatoxin biosynthesis, analysis of the activity measured in mycelia samples cultivated in PDBUU for 24 h demonstrated that the catalase activity was inhibited in the ∆*sfgA* mutant at 30 °C while it was increased at 36 °C compared with the control strain (Figure 5E). This result indicated that *sfgA* would be involved in intracellular oxidative stress balance and takes part in regulating aflatoxin biosynthesis in *A. flavus*.

In addition, the production of kojic acid, an important chemical material utilized to manufacture various cosmetics and pharmaceutics, was positively affected by *sfgA* at both temperatures under all media tested (Figure 5F and Supplementary Figure S4). The aforementioned results indicated that *sfgA* exerts a vital and complex role in *A. flavus* secondary metabolite biosynthesis.

### 3.5. The Effect of sfgA on Response to Environmental Stress

**Figure 6:**
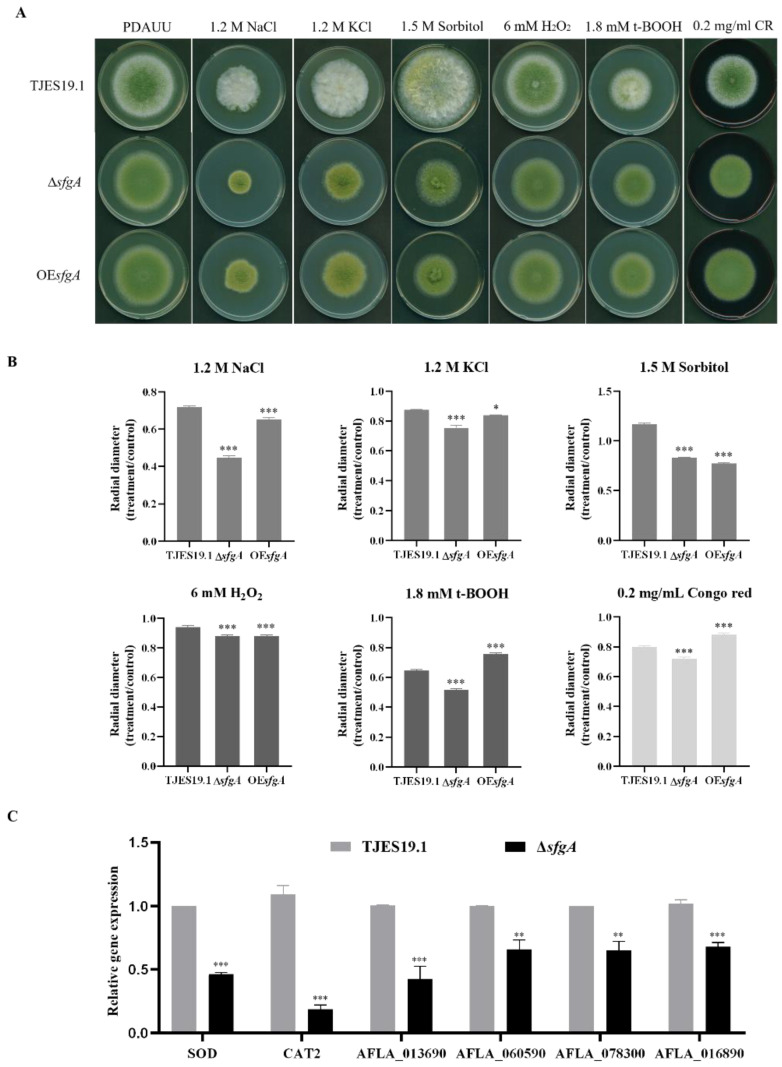
Phenotypes of the ∆*sfgA* mutant in various stress conditions. (**A**) TJES19.1, ∆*sfgA*, and OE*sfgA* strains were point-inoculated on solid PDAUU media containing various compounds including NaCl, KCl, sorbitol, H_2_O_2_, t-BOOH, and CR at 30 °C for 5 days. (**B**) Quantitative analysis of colony diameter shown in (**A**). (**C**) qPCR analysis of transcriptional levels of oxidative and cell-wall-related genes of mycelia cultured on PDAUU at 30 °C for 24 h. Gene expression levels at each time point were normalized to *β*-actin by 2^−ΔΔ^CT analysis. * *p* ≤ 0.05; ** *p* ≤ 0.01; *** *p* ≤ 0.001.

Control and mutant strains were inoculated on PDAUU with several compounds that are related with osmotic stress (NaCl, KCl, and sorbitol), oxidative stress (H_2_O_2_ and t-BOOH), and cell wall stress (Congo red). The ∆*sfgA* mutant strain displayed more sensitivity to all stressors tested and could produce more conidia under various environmental stress than the control strain (Figure 6A,B), which indicated that the deletion of *sfgA* conferred *A. flavus* more sensitivity to various stress conditions.

In addition, we detected the relative expression of two ROS scavenging enzymes (SOD and CAT2) encoding genes, three cell-wall-related genes (AFLA_013690, AFLA_060590, and AFLA_078300, which encode different chitin synthases), and one cell wall integrity regulator gene, AFLA_016890. As shown in Figure 6C, the transcriptional levels of all of the above genes in the ∆*sfgA* strain were significantly lower than those in the control strain. These results suggest that the response of *sfgA* to environmental stress resistance may be through affecting the cell wall integrity and intracellular oxidative stress balance.

### 3.6. Transcriptome Analysis of the ∆sfgA strain

**Figure 7:**
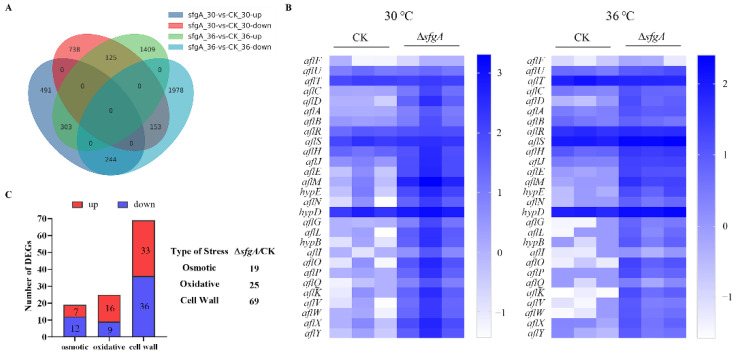
Transcriptome analysis of *sfgA* in *A. flavus*. (**A**) Venn diagram of common and differentially expressed gene number among different comparison groups. (**B**) Heat map showing transcript abundance of genes of the parental and Δ*sfgA* strains associated with aflatoxin production at 30 °C and 36 °C, respectively. The heat map is color-coded and represents the log10 value of the FPKM values of each gene in ∆*sfgA* and control samples. (**C**) Impact of *sfgA* on the expression of stress-related genes in *A. flavus* at 30 °C.

To investigate how *sfgA* affected the overall gene expression in *A. flavus*, RNA sequencing analysis was carried out between the *sfgA* deletion mutant (named group *sfgA*) and the TJES19.1 strain (named group CK) cultured at 30 °C and 36 °C for 48 h on GMMUU media. The results are highly reproducible and reliable (Supplementary Table S3 and Supplementary Figure S5). Alignments were prepared with DESeq2 [30] for a genome-wide analysis of differential gene expression. When propagated at 30 °C, the expression of 1038 genes in the ∆*sfgA* strain (sfgA-30) were significantly up-regulated, while the expression of 1016 genes were down-regulated compared with the TJES19.1 strain (CK-30) (fold change ≥ 2, *q*-value ≤ 0.05). When cultured at 36 °C, the expression of 1837 genes in the ∆*sfgA* strain (sfgA-36) were clearly up-regulated, while the expression of 2375 genes were down-regulated compared to the TJES19.1 strain (CK-36) (fold change ≥ 2, *q*-value ≤ 0.05) (Figure 7A).

The transcriptional activities of genes involved in fungal development are shown in Supplementary Table S4. The *brlA*, *con-6*, *con-10*, and *rodB* genes related to conidiation in the ∆*sfgA* strain were up-regulated at both 30 °C and 36 °C, which is consistent with the result that showed the ∆*sfgA* strain produced more conidiophores. Remarkably, the genes encoding the Cys_2_His_2_ (C_2_H_2_) zinc finger transcription factor NsdC and the sexual development transcription factor NsdD were up-regulated at 30 °C but down-regulated at 36 °C. Moreover, the expression of the *ppoA* gene which induced sexual reproduction was also up-regulated at 30 °C and down-regulated at 36 °C. Additionally, the expression of the *ppoC* gene which induced asexual development was down-regulated at 30 °C and exhibited no variation at 36 °C. These data may provide some clues for the contradicting phenotypes of sclerotial number in the ∆*sfgA* strain cultured at different temperatures.

As to the transcriptional changes in secondary metabolism genes induced by *sfgA* deletion, it was found that 42 out of the predicted 56 secondary metabolite gene clusters [31,32,33] were differentially expressed, including the asparasones cluster (#27), aflavarins cluster (#39), aflatoxin cluster (#54), and kojic acid cluster (#56) (Supplementary Table S5). For example, at least 24 out of the 34 aflatoxin cluster genes were significantly up-regulated in the ∆*sfgA* strain at both temperatures (Figure 7B), which was consistent with the result of aflatoxin detection. Additionally, *kojA* (AFLA_096040) in the kojic acid cluster was obviously down-regulated in the ∆*sfgA* strain cultured on GMMUU media at 30 °C.

After analyzing the expression of genes involved in the environmental stresses, we found that the absence of *sfgA* resulted in the significant deviation of the expression levels of approximately 113 genes related to stress response from the control strain (Figure 7C and Supplementary Table S6).

## 4. Discussion

Previous studies have shown that SfgA in *A. nidulans* is a negative regulator of conidiation, functioning downstream of FluG but upstream of other key developmental activators, including FlbD, FlbC, FlbB, and BrlA, which are necessary for normal conidiation and sterigmatocystin biosynthesis [25]. In this paper, we found that *sfgA* in *A. flavus* has broad regulatory roles, including in growth, conidiation, sclerotia formation, secondary metabolism, and environmental stress responses. *sfgA* exhibits differential effects in sclerotia production at different temperatures. *sfgA* in *A. flavus* also displays the regulation of environmental stress responses and secondary metabolism in a complex way. Our research indicated that the regulatory function of the *sfgA* gene in *A. flavus* may be alterable with changes in external environmental factors, which was further illuminated via a comparative transcriptomic study of ∆*sfgA* mutant.

The formation of conidia in *A. flavus* requires the concerted activity of a number of signaling proteins and transcription factors. For example, the *brlA* gene encodes a Cys_2_His_2_ (C_2_H_2_) zinc finger transcription factor which regulates the developmental switch from vegetative cells to conidiophores [14]. *con-6*, together with *con-10*, is involved in desiccation stress and conidial germination in *A. nidulans* [34]. Based on our experimental data, *sfgA* in *A. flavus* could negatively influence the conidia formation, which was consistent with the result in *A. nidulans* [25]. We also found that the transcription levels of the conidia-specific genes *brlA*, *con-6*, *con-10*, and *rodB/hypB* were up-regulated significantly, but the transcription levels of the *fluG* and *flbs* genes related to mycelia development exhibited no difference when the *sfgA* deletion mutant of *A. flavus* was propagated at 30 °C, which indicates that the *sfgA*-mediated repression of the conidia formation in *A. flavus* may be realized by affecting the expression of conidiophore development genes but not by altering the expression of *flbs*. These results were distinct from those in *A. nidulans* on the mechanism of conidia development [18,25].

Sclerotia is a sexual structure for survival under harsh environments in *A. flavus* [4]. Sexual reproduction in fungi requires the presence of many specific genes in the genome. In this study, we found that the sclerotia formation of the ∆*sfgA* mutant was significantly different from the control strain when cultured at both 30 °C and 36 °C. This difference was recorded in the sclerotia amount and the expression of the regulating genes *nsdC* [35] and *nsdD* [36], which were essential for sclerotia production. The differential expression of *ppoA* and *ppoC* genes, which both encode putative fatty acid oxygenases, can balance sexual and asexual spore development [37,38]. The deletion of *ppoA* in *A. nidulans* resulted in a fourfold rise in the ratio of asexual to sexual spore amounts due to a decrease in psiBα levels (precocious sexual inducer). The over-expression of *ppoA*, on the other hand, resulted in elevated levels of psiBα and a sixfold reduction in the ratio of asexual to sexual spore amounts [39]. An increased ratio of sexual to asexual spore amounts was also observed after the deletion of *ppoC* [39]. Alterations in the sexual sclerotia production in the ∆*sfgA* mutant at 30 °C and 36 °C were concomitantly reflected in mRNA levels of *ppoA* and *ppoC* genes in *A. flavus*. The deletion of *sfgA* increased sexual sclerotial numbers with the up-regulated expression of the *ppoA* gene as well as down-regulated *ppoC* gene expression at 30 °C. What is interesting is that the deletion of *sfgA* decreased the sclerotial numbers with the down-regulated expression of the *ppoA* gene at 36 °C, while the expression of the *ppoC* did not display any variation. Thus, it is clear that *sfgA* of *A. flavus*, in our study, plays different roles in sexual sclerotia production by affecting the expression of *ppoA* and *ppoC* genes.

In addition, the change in secondary metabolite production was correlated with conidiophore formation and sclerotia production [40]. In *A. flavus*, several genetic co-regulators, which activate the genes involved in secondary metabolite production and the formation of spores and sclerotia, were identified [41]. For example, in the *A. flavus ∆veA* strain, more conidia but no sclerotia were produced, and more importantly, *veA* was required for the production of aflatoxin, cyclopiazonic acid, and asparasone, which have been isolated from the sclerotia [42]. In our study, we found that *sfgA* regulated AFB1 biosynthesis in a complex way in response to the changes in culture conditions (Figure 5A–C). Additionally, through the qPCR (Figure 5D) and RNA-seq (Figure 7B) analyses, we found that the crucial regulator gene *aflR* was slightly activated, accompanied with significant activation in the expression of aflatoxin structural genes, leading to a consecutive increase in the ability to synthesize aflatoxin and its intermediates when cultured in PDBUU and GMMUU media at 30 °C, which suggests that *sfgA* regulated aflatoxin biosynthesis by affecting the aflatoxin cluster genes. It has been reported that ROS induces aflatoxin synthesis, and catalase can remove ROS to protect cells from oxidative stress [43,44]. In our experiment, we also found that catalase activity changed at different temperatures. The loss of the *sfgA* gene resulted in different aflatoxin production levelsat different temperatures, which may have been caused by changes in the ROS clearance system in *A. flavus*. In fact, aflatoxin production is a very complex process and is susceptible to external factors [45]. Temperature is one of the most important factors affecting growth and aflatoxin biosynthesis in *A. flavus*. A number of studies have reported that temperature may affect the expression of aflatoxin cluster structural genes by regulating the specific regulatory factors AflR and AflS, leading to changes in aflatoxin biosynthesis [46]. So far, other transcription factors or related receptors that regulate the response to temperature change have not been found, and how to transmit the signals of temperature change is still unknown. Thus, the observations made regarding *sfgA* can be insightful. Furthermore, aflatoxin production is also influenced by nutritional conditions, including carbon sources, amino acids, trace elements, pH, and so on [41,47], and the effect of pH depends on the composition of the medium [48]. Taken together, *sfgA* plays a complex role in aflatoxin production.

In addition, the size of sclerotia produced by the Δ*sfgA* mutant was much smaller compared to the control strain when propagated at 30 °C, which is consistent with the research reported by Chang et al. [29]. They described that the increase in toxin production coincided with a decrease in sclerotia size and an alteration in sclerotia shape, together with an increase in sclerotial numbers in some cases, and they suggested that these alterations could be caused by competition for a common substrate such as acetate. Our result confirmed that *sfgA* in *A. flavus* is a co-regulator of the secondary metabolism and sclerotia production, which is similar to the function of *A. flavus aswA* which regulates sclerotial development and the biosynthesis of sclerotium-associated secondary metabolites [49].

*sfgA* plays a major role in the secondary metabolism. Apart from aflatoxin, dozens of other secondary metabolism gene clusters, including kojic acid, asparasones, and aflavarins, were influenced by deleting *sfgA* in *A. flavus* according to our transciptome data. Our kojic acid detection results confirmed the positive regulation of *sfgA* on kojic acid formation in *A. flavus*, and the transcription level of *kojA* involved in the kojic acid biosynthesis pathway was also down-regulated in the ∆*sfgA* mutant. In fact, different secondary metabolic pathways are usually co-regulated to maintain cellular homeostasis and promote cell survival under stress conditions [50].

We also found that the deletion of *sfgA* increased the sensitivity of *A. flavus* when the ∆*sfgA* mutant was challenged by osmotic, oxidative, and cell wall stresses, which was confirmed with the transcriptome result shown in Supplementary Table S6. The RNA-seq data demonstrated that some of differently expressed genes in the MAPK pathway [51] play a pivotal role in the osmotic stress response in *Aspergillus*. These genes were consistently down-regulated, including sensor histidine kinase TcsB, MAP kinase kinase Ste7, Ste20-like serine, protein tyrosine phosphatase Pps1, and Mst3-like protein kinase (Supplementary Table S6). Our finding suggested that *sfgA* responses to the osmotic pressure may occur through the MAPK pathway.

Some studies have found that five complexes (I~V) are involved in oxidative stress and phosphorylation [52,53]. As shown in Supplementary Figure S6, our RNA-seq data showed that the expressional levels of some genes encoding NADH dehydrogenase, succinate dehydrogenase, cytochrome oxidase, and ATPase in the complexes were down-regulated to different degrees in the ∆*sfgA* mutant, which suggests that *sfgA* compromises fungal oxidative stress tolerance, which maybe mediated by altering mitochondrial respiration [54].

The cell wall is not only essential for the survival of fungi during development and reproduction, but it also acts as a protective barrier for fungi against environmental factors [55]. According to our RNA-seq data, the transcript of chitin synthase gene *chs3* [56] was moderately down-regulated in the ∆*sfgA* mutant. The regulatory subunit of the *rho* family of GTPases is essential to the cell wall integrity signaling pathway, and it has been confirmed that the deletion of the *rho* protein resulted in cytoplasmic leakage in *Aspergillus fumigatus* [57]. In our study, the Rho GTPase activator Lrg11 was down-regulated in the *sfgA* deletion mutant, and the important component of fungal cell wall, the alpha−1,3-glucan synthase encoded by *ags*1 and *ags*3 [58], was also significantly up-regulated in the *sfgA* deletion mutant. These results suggested that the deletion of *sfgA* may affect the main components of the fungal cell wall of *A. flavus*, including chitin and structural polysaccharides. Consequently, the cells will generate a defensive response to by over-expressing alpha-1,3-glucan synthase genes to overcome stimulation [59]. In general, it is possible that the response of *sfgA* to environmental stress resistance could be linked to the differential expression of these genes.

## 5. Conclusions

In this study, we explored the diversified roles of the *sfgA* gene in fungal pathogen *A. flavus*. We verified that *sfgA* can regulate the growth, conidiation, sclerotia formation, secondary metabolism, and environmental stresses responses in *A. flavus* in a complex way. Our findings shed light on the roles of *sfgA* in the regulatory mechanisms of morphogenesis and the secondary metabolism in filamentous fungi.

## Data Availability

Data is contained within the article or Supplementary Materials.

## Supplementary Materials

The following supporting information can be downloaded at: https://www.mdpi.com/article/10.3390/jof8060638/s1, Table S1: Primers used for constructing *sfgA* deletion and over-expression strains; Table S2: Primers used for qPCR; Table S3: Reads and reference genome comparison; Table S4: Transcript abundance of genes involved in *A. flavus* development; Table S5: Transcript abundance of genes that are involved in secondary metabolism; Table S6: Transcript abundance of genes that are involved in environmental stresses; Figure S1: PCR confirmation of the *sfgA* deletion (A, B, C) and over-expression (D, E, F) transformants; Figure S2: *sfgA* affects conidia growth and spore germination of *A. flavus*; Figure S3: Phenotypes of ∆*sfgA*∆*fluG* in *A. flavus*; Figure S4: Determination of kojic acid production in solid YGTUU and YESUU medium for 36 h via colorimetric method; Figure S5: Overview of RNA-seq results; Figure S6: The diagram of oxidative phosphorylation including complexes I, II, III, IV, and V.
