# Supplementary material for: SfgA Renders Aspergillus flavus More Stable to the External Environment
Source: J Fungi (Basel). 2022 Jun 16;8(6):638. doi: 10.3390/jof8060638 (PMC9224668; doi:10.3390/jof8060638)
Supplement: Supplementary file 1 [file jof-08-00638-s001.zip › jof-1738709-supplementary.pdf]

Supplementary Materials

Table S1. Primers used for constructing *sfgA* deletion and over-expression strains.

Name	Sequence
P1	GCTATGCCCAAAGGGTTCG
P2	CGAACTCATCACCACCGGGAGGGACGTAGATATGTCGGAGGA
P3	TCCCGGTGGTGATGAGTTCG
P4	CCCGTGACATGTGAATGCGG
P5	CCGCATTCACATGTCACGGGTCGTCTACTACCCATACAACCTCCC
P6	CATTCTGAACATCATCTGGCTTCC
P7	GGCGTGAATCCTTTTGAGACC
P8	GGAGCAAGCCGTTCCAGTTC
P9	GCTATGCCCAAAGGGTTCGGGATCT
P10	GAGGTGAGATGGGATTGGTATAGGA
P11	TCCTATACCAATCCCATCTCACCTCTCCCGGTGGTGATGAGTTCGGTAGA
P12	CCCGTGACATGTGAATGCGGGCACC
P13	GGTGCCCGCATTCACATGTCACGGGCATCCGGATGTCGAAGGCTTGGGGC
P14	CATTGTGATGTCTGCTCAAGCGGGG
P15	CCCCGCTTGAGCAGACATCACAATGATGGAGATCCAAACGGTAGTCTGCG
P16	TCACGGAAGGAGAATCGAAGACCGA
P17	TCGGCGTGAATCCTTTTGAGACCACA
P18	GAGTAGACGATATATCTCACGAGCACCT
P19	TTGTTATTCAGAGCGTGGGACTAAGG
P20	AGAAGTAGCAGTGTGAGAAGTGTATCG
P21	GGCTTCCAGATCACTTCGGAACTTG
P22	AAGAACACCGCAATCGCAATCGTA
P23	CGGATGACTGTCGAACTGCATGATAGAG
P24	AGAACACCGCAATCGCAATCGTAGC
P25	ACTAGATAGTAGACAGGTAGGACACGAT
P26	AGAGTCAACAGCAGCAACAAGAGA
P27	CCAAATATCGTGCCTCTCCTGCTTT
P28	GCTTGAATCTCCATCATCACTGACCAA
P29	CTGTGCAAGATGTTCTCGAATGCCGCTA
P30	CTCGATACAGCCCGCATATTCCAAGACC

Table S2. Primers used for qPCR.

Name	Sequence
actin-F	AGGACTCTTATGTCGGTGATG
actin-R	CGGTTGGACTTAGGGTTG
sfgA-F	CGCCAAGCCGAAATGAACCAC
sfgA-R	GTTATATTCCCAGCCGCCACC
aflC-F	TGTCAGACCACAAACGCACCT
aflC-R	CATCTCACAGAACGCCCTCAA
aflD-F	CGCCTGAGGAGACGGTGTATT
aflD-R	CTGCCTTCAGCGACGGTTAG
aflB-F	TCAAGCCCGTACCATTCATCC
aflB-R	ATTACCGCCTCCACATCTTCG
aflA-F	CCTCCCTCTGATGCCGGTGA
aflA-R	ATTTCAATGCAGCGACCCGAT
aflR-F	GCAGTCAATGGAACACGGAAAC
aflR-R	CCTGAAACGGTGGTAGTGGG
aflS-F	CTGACCATCTCCGACCCGTTC
aflS-R	AGAGCCAACTGTCGGACCAA
aflH-F	CACCCCACTGATTGCGCCTA
aflH-R	ATCGCCTCCCCAACCATACTCG
aflJ-F	CTGCGTTGCTACACTCCCC
aflJ-R	ATCACGCGGCAGAAACCATC
aflE-F	ACAGAAGAACACCAAGGCTAC
aflE-R	CGGATTTCCTCCTCGCTCAG
aflM-F	CCGTTTAGATGGCAAAGTGGC
aflM-R	TCACGGGAATGGGCGTAGTT
aflN-F	ACCTTCCTATGCCAGAGTTCA
aflN-R	AATCATCCAGGTCCCGCTA
aflG-F	GACTCGTTCGGCTGCCTTGA
aflG-R	GTTCCGGCGTCGCTGTTCTA
aflL-F	CCGACAAGTTCATCCCCGAG
aflL-R	TGCGCCCAATACAGTTCCG
aflI-F	CTTCGCATTCAAATCCTCGTT
aflI-R	CGCACATGAACACAATACTAGCA

Table S3. Reads and reference genome comparison.

Sample	Total Reads	Mapped Reads	Mapping Rate (%)
CK30_1	49,725,142	47,130,952	94.78
CK30_2	46,968,204	43,200,080	91.98
CK30_3	41,459,326	40,064,515	96.64
CK36_1	46,454,732	43,897,365	94.49
CK36_2	47,044,448	45,566,330	96.86
CK36_3	42,395,790	40,298,429	95.05
*sfgA*30_1	44,333,636	38,603,367	87.07
*sfgA*30_2	45,902,894	40,878,091	89.05
*sfgA*30_3	45,401,428	39,258,303	86.47
*sfgA*36_1	46,551,820	40,046,252	86.03
*sfgA*36_2	46,339,332	40,957,455	88.39
*sfgA*36_3	46,401,238	42,469,803	91.53

Table S4. Transcript abundance of genes involved in *A. flavus* development.

Gene ID	Name	Function	Δ*sfgA* vs. Control Log2 Fold Change		Up/Down	
			30 °C	36 °C	30 °C	36 °C
AFLA_082850	*brlA*	C_2H_2 type master regulator of conidiophore development	1.5525	2.0604	up	up
AFLA_044800	*con-6*	Conidiation- protein	1.6801	1.5497	up	up
AFLA_083110	*con-10*	Conidiation-specific protein	1.6946	1.9740	up	up
AFLA_014260	*rodB*	Conidial hydrophobin	2.8596	2.4726	up	up
AFLA_131330	*nsdC*	Sexual development transcription factor	1.5707	−1.2657	up	down
AFLA_020210	*nsdD*	Sexual development transcription factor	1.9961	−1.2916	up	down
AFLA_026790	*ppoA*	Balance sexual and asexual development	1.6422	−5.4199	up	down
AFLA_030430	*ppoC*	Balance sexual and asexual development	−2.8073	ns	down	ns

Log_2 Fold Change ≥ 1 indicates up-regulated expression and Log_2 Fold Change ≤ −1 indicates down-regulated expression. *q*-value < 0.05. ns: no significance

Table S5. Transcript abundance of genes that are involved in secondary metabolism (fold change ≥ 2, p-value ≤ 0.05).

Cluster	Gene name [a]	Gene Description	$Log_2FoldChange$
1	**AFLA_002900**	polyketide synthase, putative	−1.207276
2	AFLA_004270	conserved hypothetical protein	1.647357
3	AFLA_004440	ABC multidrug transporter, putative	1.134126
3	AFLA_004450	nonribosomal peptide synthase, putative	3.089120
4	**AFLA_005440**	nonribosomal peptide synthase, putative	1.506663
4	AFLA_005450	conserved hypothetical protein	4.177949
4	AFLA_005460	cytochrome P450, putative	1.949813
6	AFLA_008760	multidrug resistance protein, putative	2.019229
7	AFLA_009120	NRPS-like enzyme, putative	1.836413
7	AFLA_009130	NRPS-like enzyme, putative	1.836413
7	**AFLA_009200**	conserved hypothetical protein	−1.714063
8	**AFLA_010020**	nonribosomal peptide synthase, putative	4.310527
9	AFLA_010570	hypothetical protein	−1.131355
9	**AFLA_010580**	nonribosomal peptide synthase, putative	2.205267
11	AFLA_022990	conserved hypothetical protein	1.562315
11	AFLA_023000	Ankyrin domain protein	−2.669890
11	AFLA_023040	C6 transcription factor, putative	−1.987002
11	AFLA_023050	MFS transporter, putative	−3.380049
11	AFLA_023070	integral membrane protein	−2.954750
12	**AFLA_028720**	NRPS-like enzyme, putative	−2.474905
15	AFLA_045450	ankyrin repeat-containing protein, putative	1.924687
15	AFLA_045560	carboxylic acid transport protein	−2.047874
15	AFLA_045570	acetyl xylan esterase, putative	−4.587482
16	**AFLA_047190**	L-ornithine N5-oxygenase SidA	−1.786393
17	AFLA_053840	conserved hypothetical protein	3.604693
19	AFLA_060670	integral membrane protein	−1.600693
19	**AFLA_060680**	dimethylallyl tryptophan synthase, putative	−4.745230
19	AFLA_060690	cytochrome P450, putative	−3.827991
19	AFLA_060700	phenylalanine ammonia-lyase, putative	−1.863934

19	AFLA_060710	toxin biosynthesis protein, putative	−2.594664
20	AFLA_062800	aldo-keto reductase, putative	2.183065
20	AFLA_062840	conserved hypothetical protein	1.668746
21	AFLA_064410	conserved hypothetical protein	−2.760077
21	AFLA_064430	macrolide phosphotransferase k, putative	−1.653123
21	AFLA_064500	conserved hypothetical protein	−2.026403
21	AFLA_064570	uracil permease, putative	2.037579
22	AFLA_066690	pyrroline-5-carboxylate reductase, putative	1.651149
22	AFLA_066700	P450 family sporulation-specific N-formyltyrosine oxidase Dit2	1.568090
22	**AFLA_066720**	conserved hypothetical protein	−2.026403
23	**AFLA_066840**	hybrid PKS/NRPS enzyme, putative	1.399046
23	AFLA_066880	monocarboxylate transporter, putative	4.224464
23	AFLA_066910	conserved hypothetical protein	3.939787
23	AFLA_066920	alcohol dehydrogenase, putative	4.214583
23	AFLA_066940	O-methyltransferase, putative	4.005561
23	AFLA_066950	conserved hypothetical protein	−3.512477
23	AFLA_066970	conserved hypothetical protein	−3.972694
23	**AFLA_066980**	polyketide synthase, putative	−2.918739
24	AFLA_069320	ABC multidrug transporter, putative	−2.031826
25	AFLA_070870	isopenicillin N synthetase PcbC	−2.421427
25	AFLA_070880	acyl-coenzyme A:Isopenicillin N acyltransferase PenDE	−4.390697
26	**AFLA_079400**	NRPS-like enzyme, putative	−1.003081
27	AFLA_082160	MFS glucose transporter, putative	−2.611276
28	AFLA_082460	conserved hypothetical protein	1.682483
30	AFLA_090170	MFS sugar transporter, putative	−2.436712
30	AFLA_090210	amino acid permease, putative	−1.729707
30	AFLA_090220	integral membrane protein	−1.129052
31	AFLA_095020	hypothetical protein	1.169802
31	AFLA_095060	tyrosinase, putative	1.258638
31	AFLA_095100	conserved hypothetical protein	1.276701
31	AFLA_095120	efflux pump antibiotic resistance protein, putative	−1.224329
32	AFLA_096410	PTH11-like integral membrane protein, putative	2.089426
34	AFLA_100290	aliphatic nitrilase, putative	1.319157
34	AFLA_100310	oxidoreductase, putative	2.520110
37	AFLA_105170	O-methyltransferase, putative	1.706003

37	**AFLA_105190**	NRPS-like enzyme, putative	1.363957
39	AFLA_108540	NADH oxidase, putative	1.233473
41	**AFLA_114820**	polyketide synthase, putative	2.792072
42	AFLA_116180	hypothetical protein	1.369392
42	AFLA_116210	O-methyltransferase, putative	1.488287
43	AFLA_116510	conserved hypothetical protein	1.460287
43	AFLA_116560	NmrA-like family protein	−1.486997
43	AFLA_116570	DUF636 domain protein	−1.522362
43	AFLA_116630	(S)-2-hydroxy-acid oxidase, putative	1.919610
44	AFLA_116920	short-chain dehydrogenase, putative	−1.248762
45	AFLA_118390	NCS family nucleoside transporter, putative	−3.269836
45	AFLA_118450	conserved hypothetical protein	1.127589
45	AFLA_118460	amino acid transporter, putative	−3.014212
46	**AFLA_118940**	polyketide synthase, putative	3.272329
46	AFLA_118950	conserved hypothetical protein	4.115391
46	**AFLA_118960**	polyketide synthase, putative	3.685196
47	AFLA_119120	beta-lactamase family protein	−1.743214
48	**AFLA_121520**	NRPS-like enzyme, putative	1.710782
48	AFLA_121530	NADH-dependent flavin oxidoreductase, putative	1.335394
48	AFLA_121590	zinc-binding alcohol dehydrogenase, putative	2.055708
48	AFLA_121630	short-chain dehydrogenase/oxidoreductase, putative	2.841643
51	AFLA_127110	MFS transporter, putative	−1.071045
52	**AFLA_128060**	polyketide synthase, putative	1.633030
52	AFLA_128070	conserved hypothetical protein	1.920275
52	AFLA_128080	conserved hypothetical protein	1.524287
53	AFLA_135470	ornithine decarboxylase, putative	3.899490
53	AFLA_135480	galactose-proton symport, putative	2.657110
54	AFLA_139150	aflY/ hypA/ hypP/ hypothetical protein	7.003634
54	AFLA_139160	aflX/ ordB/ monooxygenase/ oxidase	6.215823
54	AFLA_139170	aflW/ moxY/ monooxygenase	7.080860
54	AFLA_139180	aflV/ cypX/ cytochrome P450 monooxygenase	6.721848
54	AFLA_139190	aflK/ vbs/ VERB synthase	6.415022
54	AFLA_139200	aflQ/ ordA/ ord-1/ oxidoreductase/	6.881341

		cytochrome P450 monooxigenase	
54	AFLA_139210	aflP/ omtA/ omt-1/ O-methyltransferase A	7.308268
54	AFLA_139220	aflO/ omtB/ dmtA/ O-methyltransferase B	6.955609
54	AFLA_139230	aflI/ avfA/ cytochrome P450 monooxygenase	8.764194
54	AFLA_139240	aflLa/ hypB/ hypothetical protein	7.369943
54	AFLA_139250	aflL/ verB/ desaturase/ P450 monooxygenase	7.234853
54	AFLA_139260	aflG/ avnA/ ord-1/ cytochrome P450 monooxygenase	7.118147
54	AFLA_139270	aflNa/ hypD/ hypothetical protein	1.478176
54	AFLA_139280	aflN/ verA/ monooxygenase	5.581364
54	AFLA_139290	aflMa/ hypE/ hypothetical protein	5.833527
54	AFLA_139300	aflM/ ver-1/ dehydrogenase/ ketoreductase	8.132586
54	AFLA_139310	aflE/ norA/ aad/ adh-2/ NOR reductase/ dehydrogenase	6.655786
54	AFLA_139320	aflJ/ estA/ esterase	4.996253
54	AFLA_139330	aflH/ adhA/ short chain alcohol dehydrogenase	2.726443
54	AFLA_139370	aflB / fas-1 / fatty acid synthase beta subunit	3.413627
54	AFLA_139380	aflA / fas-2 / hexA / fatty acid synthase alpha subunit	4.018693
54	AFLA_139390	aflD / nor-1 / reductase	7.987878
54	AFLA_139400	aflCa / hypC / hypothetical protein	7.974146
54	AFLA_139410	aflC / pksA / pksL1 / polyketide synthase	4.605925
54	**AFLA_139360**	aflR / apa-2 / afl-2 / transcription activator	1.002267
56	AFLA_096040	conserved hypothetical protein	−1.927421

[a] The bold genes represent backbone genes in each cluster.

Table S6. Transcript abundance of genes that are involved in environmental stresses (fold change ≥ 2, p-value ≤ 0.05).

Gene name	Gene Description	$Log_2FoldChange$	Stress
AFLA_010860	F-box and wd40 domain protein, putative	0.3852646	osmotic
AFLA_019280	peroxiredoxin, putative	0.289199752	osmotic
AFLA_022380	molecular chaperone Hsp70	0.487892937	osmotic
AFLA_024540	sensory transduction histidine kinase, putative	2.450629368	osmotic
AFLA_028490	conserved hypothetical protein	2.107462659	osmotic
AFLA_030340	RING finger domain protein, putative	0.086038116	osmotic
AFLA_045750	antigenic mitochondrial protein HSP60, putative	0.403137136	osmotic
AFLA_052860	chaperone/heat shock protein Hsp12, putative	2.836924545	osmotic
AFLA_086540	glycine-rich RNA-binding protein, putative	0.349908569	osmotic
AFLA_091060	allergen Asp F3	3.088393794	osmotic
AFLA_095980	mannosylphosphate transferase (Mnn4), putative	2.905110109	osmotic
AFLA_104660	sensory transduction histidine kinase, putative	2.44339302	osmotic
AFLA_108790	aldehyde dehydrogenase AldA, putative	0.202696334	osmotic
AFLA_109500	2-deoxyglucose-6-phosphate phosphatase, putative	10.31545422	osmotic
AFLA_004050	Mst3-like protein kinase, putative	0.44047059	osmotic
AFLA_039130	sensor histidine kinase/response regulator TcsB/Sln1, putative	0.194793268	osmotic
AFLA_073630	Ste20-like serine/threonine protein kinase, putative	0.424266974	osmotic
AFLA_092700	protein tyrosine phosphatase Pps1, putative	0.216988004	osmotic
AFLA_103480	MAP kinase kinase Ste7	0.410127921	osmotic
AFLA_006830	scramblase family protein	1.380665379	oxidative
AFLA_016800	thioredoxin, putative	2.019818729	oxidative
AFLA_019280	peroxiredoxin, putative	−1.789861781	oxidative
AFLA_021100	prostaglandin G/H synthase 2/cyclooxygenase 2, pgh2/cox2, putative	2.588080405	oxidative

AFLA_026790	fatty acid oxygenase PpoA, putative	1.642179042	oxidative
AFLA_027950	formamidopyrimidine-DNA glycosylase, putative	−1.521337204	oxidative
AFLA_030430	fatty acid oxygenase PpoC, putative	−2.807324701	oxidative
AFLA_036840	6-phosphogluconate dehydrogenase Gnd1, putative	1.319244861	oxidative
AFLA_039650	NADH-cytochrome B5 reductase, putative	2.639498544	oxidative
AFLA_050220	conserved hypothetical protein	2.973096451	oxidative
AFLA_051530	methylmalonate-semialdehyde dehydrogenase, putative	−2.1724422	oxidative
AFLA_052650	hypothetical protein	−1.4368895	oxidative
AFLA_052660	hypothetical protein	−1.656113973	oxidative
AFLA_052670	RNA recognition motif (rrm) domain containing protein, putative	−1.668283749	oxidative
AFLA_052860	chaperone/heat shock protein Hsp12, putative	1.504327779	oxidative
AFLA_056500	NADH-ubiquinone oxidoreductase subunit B17.2, putative	−1.435878958	oxidative
AFLA_079910	glutathione peroxidase Hyr1, putative	2.342951718	oxidative
AFLA_080370	RNA binding protein	1.1682823	oxidative
AFLA_091060	allergen Asp F3	1.626856719	oxidative
AFLA_096210	catalase, putative	1.157181053	oxidative
AFLA_110690	cytochrome c peroxidase Ccp1, putative	−1.55709287	oxidative
AFLA_120260	nitroreductase family protein, putative	2.853295979	oxidative
AFLA_121320	conserved hypothetical protein	1.108057684	oxidative
AFLA_123710	fatty acid repression mutant protein, putative	3.068695805	oxidative
AFLA_129110	sensory transduction histidine kinase, putative	1.086115064	oxidative
AFLA_001640	glycosyl hydrolase, putative	4.14716637	cell wall
AFLA_005360	conserved hypothetical protein	1.674285503	cell wall
AFLA_014130	conserved hypothetical protein	1.707869767	cell wall
AFLA_016890	Rho GTPase activator (Lrg11), putative	−1.066861467	cell wall
AFLA_018140	mannose-6-phosphate isomerase, class I	−1.726886662	cell wall

AFLA_018750	phosphatidylinositol glycan, putative	3.333858289	cell wall
AFLA_020500	Rho-like small GTPase, putative	1.54340555	cell wall
AFLA_020890	clathrin-coated vesiclec protein (Bud7), putative	−2.363906245	cell wall
AFLA_022640	protein O-mannosyl transferase	−1.468678457	cell wall
AFLA_023340	pectinesterase precursor, putative	−3.77602232	cell wall
AFLA_023430	glycosyl transferase, putative	−2.092490598	cell wall
AFLA_023460	alpha-1,3-glucan synthase Ags1	1.415947584	cell wall
AFLA_023600	cell wall protein PhiA	2.97972861	cell wall
AFLA_024280	cell wall glycosyl hydrolase Dfg5, putative	−1.033150062	cell wall
AFLA_024540	sensory transduction histidine kinase, putative	1.293152308	cell wall
AFLA_026770	alpha-1,6-mannosyltransferase subunit, putative	−1.339398526	cell wall
AFLA_027500	mannose-1-phosphate guanylyltransferase	−2.435870023	cell wall
AFLA_028260	exo-beta-1,3-glucanase (Exg1), putative	1.238299451	cell wall
AFLA_039410	cell wall serine-threonine-rich galactomannoprotein Mp1	−2.239528857	cell wall
AFLA_042780	chitin synthase A	1.018933507	cell wall
AFLA_042830	mannose-6-phosphate isomerase, putative	1.680506312	cell wall
AFLA_044970	pectate lyase precursor, putative	−1.785286741	cell wall
AFLA_045180	NAD dependent epimerase/dehydratase, putative	1.071146872	cell wall
AFLA_050610	acid phosphatase PHOa	−3.271287478	cell wall
AFLA_052510	WSC domain protein, putative	−1.294505777	cell wall
AFLA_052810	1,3-beta-glucanosyltransferase, putative	1.391643467	cell wall
AFLA_053690	alpha-L-fucosidase 2 precursor, putative	−1.714957315	cell wall
AFLA_055980	mannosyl-oligosaccharide glucosidase, putative	−1.892672392	cell wall
AFLA_056470	conserved hypothetical protein	3.377588461	cell wall
AFLA_060780	hydrophobin family protein	−1.188748914	cell wall
AFLA_061610	24-dehydrocholesterol reductase, putative	1.747873309	cell wall
AFLA_065600	translation factor, putative	−4.144452498	cell wall

AFLA_068080	cytosolic Cu/Zn superoxide dismutase, putative	1.357719977	cell wall
AFLA_069580	DUF907 domain protein	−1.286719056	cell wall
AFLA_070170	lysosomal alpha-glucosidase, putative	−2.137576192	cell wall
AFLA_073190	ab-hydrolase associated lipase, putative	−1.074510245	cell wall
AFLA_073500	conserved hypothetical protein	−1.141994216	cell wall
AFLA_075860	conserved hypothetical protein	2.416400334	cell wall
AFLA_077590	conserved hypothetical protein	2.543761827	cell wall
AFLA_078300	chitin synthase activator (Chs3), putative	−1.637301246	cell wall
AFLA_081420	extracellular SCP domain protein Pry1, putative	1.381097562	cell wall
AFLA_081820	glycosyl hydrolase, putative	1.986700523	cell wall
AFLA_081980	ferulic acid esterase (FaeA), putative	−1.750444026	cell wall
AFLA_083360	extracellular cell wall glucanase Crf1/allergen Asp F9	1.485205255	cell wall
AFLA_085140	extracellular thaumatin domain protein, putative	2.717912754	cell wall
AFLA_086360	extracellular exo-polygalacturonase, putative	−1.665482538	cell wall
AFLA_087880	integral membrane protein	1.676984007	cell wall
AFLA_103040	protein mannosyltransferase 1	−1.879198663	cell wall
AFLA_104660	sensory transduction histidine kinase, putative	1.28888594	cell wall
AFLA_104880	glycosyl hydrolase, putative	−2.845867136	cell wall
AFLA_106450	GPI anchored CFEM domain protein	2.068224179	cell wall
AFLA_107460	mannosyltransferase PMTI	−2.029910509	cell wall
AFLA_107790	glucan 1,3-beta-glucosidase precursor, putative	3.655910935	cell wall
AFLA_114760	chitin synthase B	1.093645281	cell wall
AFLA_116700	mannan endo-1,6-alpha-mannosidase DCW1 precursor, putative	1.640087707	cell wall
AFLA_116810	alpha-1,3-glucan synthase Ags3	2.337102969	cell wall
AFLA_117050	RAC GTPase, putative	1.544198732	cell wall
AFLA_119860	pectin lyase, putative	1.778960284	cell wall
AFLA_121370	1,3-beta-glucanosyltransferase, putative	2.066169112	cell wall

AFLA_122130	mannan endo-1,6-alpha-mannosidase DCW1 precursor, putative	−1.068354774	cell wall
AFLA_122480	extracellular exo-polygalacturonase, putative	1.253653238	cell wall
AFLA_123410	GPI anchored cell wall protein, putative	1.145804622	cell wall
AFLA_126870	conserved hypothetical protein	1.259492496	cell wall
AFLA_127350	N-acetylglucosamine-phosphate mutase	−2.137037927	cell wall
AFLA_128120	cell wall glucanase, putative	1.134923862	cell wall
AFLA_129100	exo-beta-1,3-glucanase, putative	−1.252829009	cell wall
AFLA_130240	DUF907 domain protein	−1.149572683	cell wall
AFLA_134770	beta-1,6 glucan synthetase (Kre6), putative	−1.743375858	cell wall
AFLA_137460	glycosyl transferase, putative	−4.627509824	cell wall

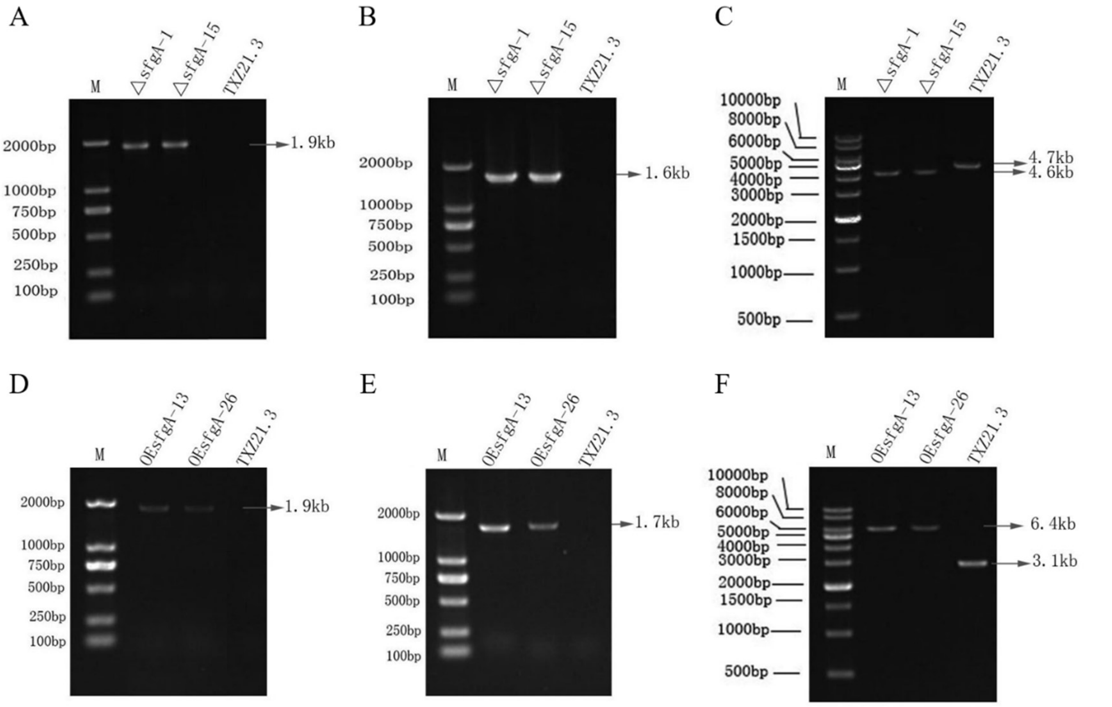

Figure S1. PCR confirmation of the *sfgA* deletion (**A**–**C**) and over-expression (**D**–**F**) transformants. (**A**) Primers P19 and P20: *sfgA* 5′ UTR - *argB*. (**B**) Primers P21 and P22: *argB* - *sfgA* 3′ UTR. (**C**) Primers P23 and P24: *sfgA* 5′ - *sfgA* 3′. (**D**) Primers P25 and P26: *sfgA* 5′ UTR -*argB*. (**E**) Primers P27 and P28: *gpdA* - *sfgA*. (**F**) Primers P29 and P30: *sfgA* 5′- *sfgA*.

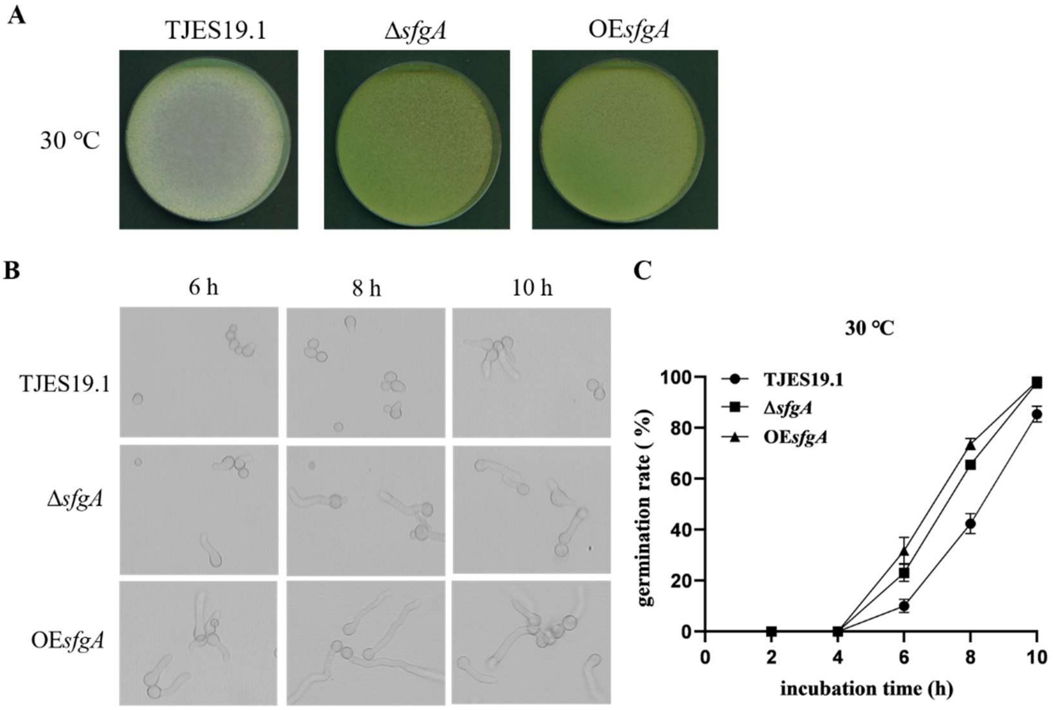

Figure S2. *sfgA* affects conidia growth and spore germination of *A. flavus*. (**A**) Conidia growth of *sfgA* mutants on PDAUU plates after 2 days incubation. (**B**) Examination of spore germination in PDBUU culture. (**C**) Comparison of germination rate of each strain.

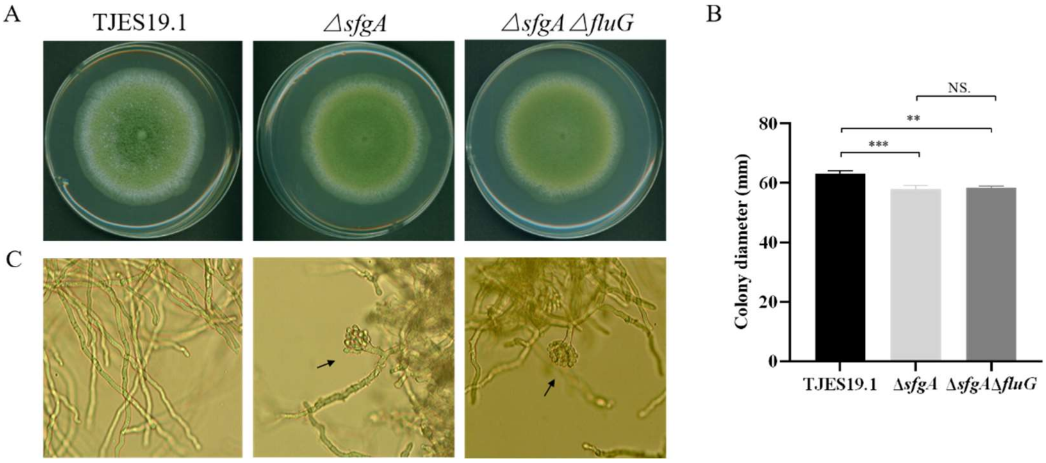

Figure S3. Phenotypes of Δ*sfgA*Δ*fluG* in *A. flavus*. (**A**) Conidia growth of control strain, Δ*sfgA* and Δ*sfgA*Δ*fluG* mutants on PDAUU plates after 5 days incubation. (**B**) Quantitative analysis of colony diameter shown in (**A**). ** $p \leq 0.01$; *** $p \leq 0.001$; NS: not significant. (**C**) Conidiophore formation in PDBUU culture was photographed at 14 h of incubation (Magnification, 200×).

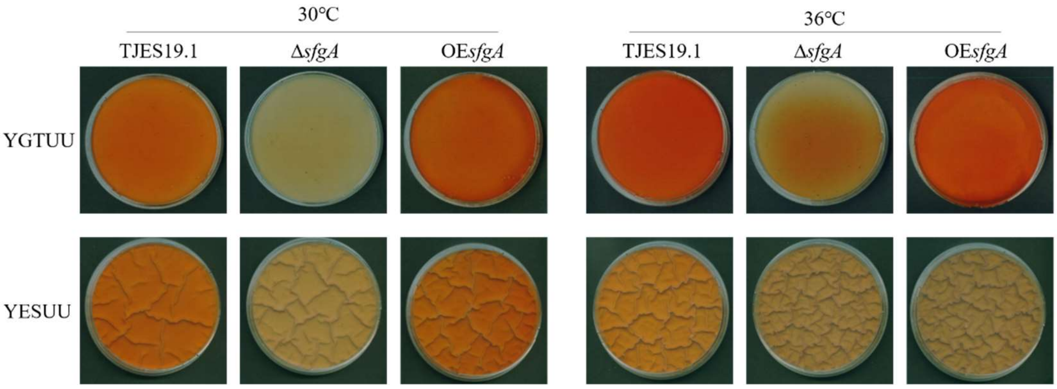

Figure S4. Determination of kojic acid production in solid YGTUU and YESUU medium for 36 h via colorimetric method.

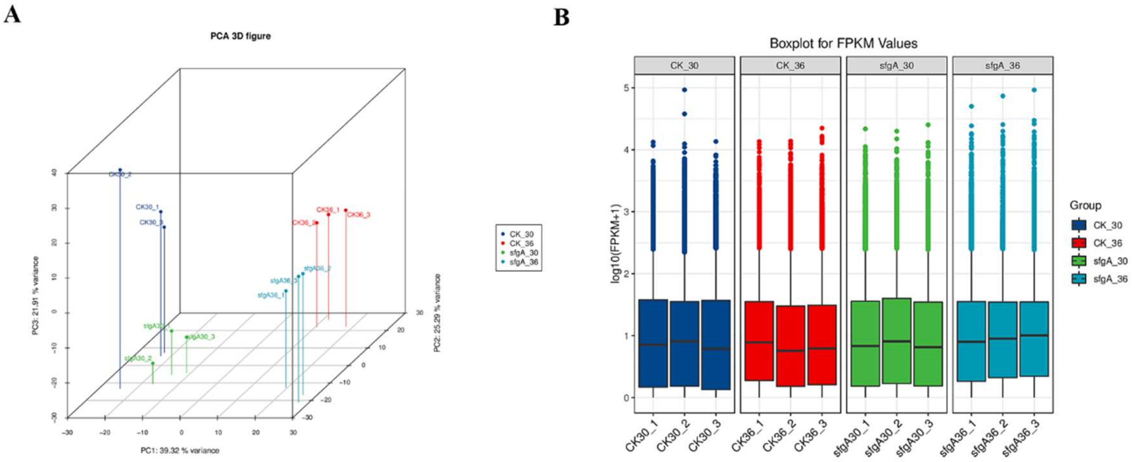

Figure S5. Overview of RNA-seq results. (**A**) PCA 3D plot of four groups. (**B**) Boxplot of overall expression levels in the four groups.

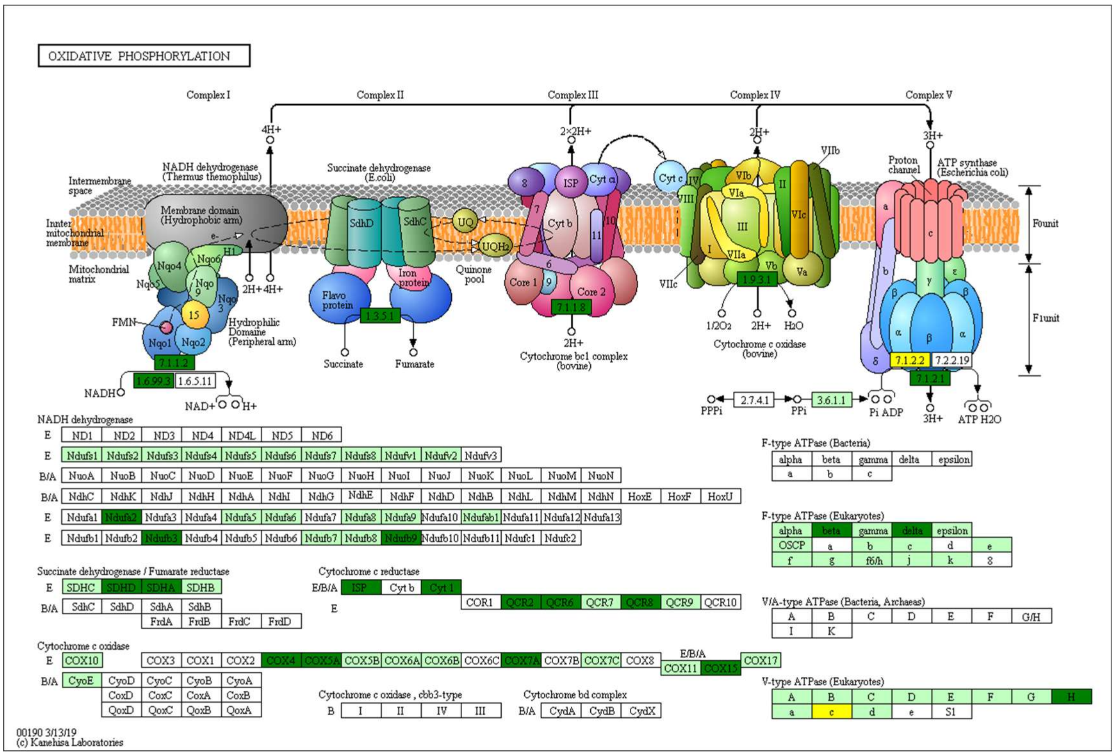

Figure S6. The diagram of oxidative phosphorylation including complexes I, II, III, IV, V. The green box represents the down-regulation expression of the gene.
